# Thermophysical properties and oxygen transport in (Th_x_,Pu_1−x_)O_2_

**DOI:** 10.1038/srep36024

**Published:** 2016-10-31

**Authors:** C. O. T. Galvin, M. W. D. Cooper, M. J. D. Rushton, R. W. Grimes

**Affiliations:** 1 Department of Materials, Imperial College London, London, SW7 2AZ, UK; 2 Materials Science and Technology Division, Los Alamos National Laboratory, P.O. Box 1663, Los Alamos, New Mexico 87545, USA

## Abstract

Using Molecular Dynamics, this paper investigates the thermophysical properties and oxygen transport of (Th_x_,Pu_1−x_)O_2_ (0 ≤ x ≤ 1) between 300–3500 K. In particular, the superionic transition is investigated and viewed via the thermal dependence of lattice parameter, linear thermal expansion coefficient, enthalpy and specific heat at constant pressure. Oxygen diffusivity and activation enthalpy are also investigated. Below the superionic temperature an increase of oxygen diffusivity for certain compositions of (Th_x_,Pu_1−x_)O_2_ compared to the pure end members is predicted. Oxygen defect formation enthalpies are also examined, as they underpin the superionic transition temperature and the increase in oxygen diffusivity. The increase in oxygen diffusivity for (Th_x_,Pu_1−x_)O_2_ is explained in terms of lower oxygen defect formation enthalpies for (Th_x_,Pu_1−x_)O_2_ than PuO_2_ and ThO_2_, while links are drawn between the superionic transition temperature and oxygen Frenkel disorder.

There is a growing and renewed interest in nuclear fuel cycles based on Th as they have some beneficial features. For instance, Th bearing ores are relatively abundant (four times more so than U^1^,^2^) meaning that such fuel cycles could extend the time scale over which nuclear energy can be generated. It has also been suggested that Th provides a more proliferation resistant path to nuclear power^1^ (although how resistant is a matter of debate^2^). Moreover, the feasibility of Th utilisation has been demonstrated in a wide variety of reactor types^1^.

In nature, Th is almost exclusively found as its fertile, but non-fissile ^232^Th isotope. Although this can be bred to give fissile ^233^U, an initial source of neutrons is required. Blending of ^232^Th with a fissile isotope (such as ^235^U or ^239^Pu) to form a mixed oxide fuel (MOX) is the most common way to achieve begining of life criticality and subsequent Th transmutation.

(Th,Pu)O_2_ is of particular interest as burning it in light water reactors (LWRs) results in a very low actinide production rate (including for Pu) and, therefore, the consumption rate of Pu is especially high^3^,^4^. This could be useful for countries such as the UK who have a large civil plutonium inventory. (U,Pu)O_2_ fuel is commonly used in LWRs, and is also a viable route for Pu burning. However, the rate of Pu destruction and the total that can be destroyed is limited since transmutation of ^238^U generates additional Pu. Th based concepts consume twice as much Pu as conventional (U,Pu)O_2_ MOX fuel^1^. The feasibility of using (Th,Pu)O_2_ fuel has been discussed for various reactor systems^5^,^6^,^7^. These studies concluded that Th based fuels can efficiently reduce Pu stockpiles while maintaining acceptable safety and control characteristics of the rector system.

One attraction of using Th in fuel is the improved thermophysical properties of ThO_2_ compared to UO_2_ and PuO_2_. For example, ThO_2_ has a higher melting point, improved thermal conductivity and lower coefficient of thermal expansion than UO_2_ and PuO_2_^1^,^8^,^9^. However, it is important to note that thermal conductivity is also dependant on phonon-defect scattering. In (U,Th)O_2_ at low temperatures there is a significant reduction in thermal conductivity, due to phonon-defect scattering from the disordered cation lattice^9^,^10^,^11^. This is also seen in (Th,Pu)O_2_^11^,^12^. As a result, understanding how the composition of these mixed oxides affects their thermophysical properties is of importance for safe and efficient reactor operation.

Using molecular dynamics (MD), this work investigates the thermal dependence of the lattice parameter, linear thermal expansion coefficient, enthalpy and specific heat at constant pressure for (Th,Pu)O_2_ systems between 300 and 3500 K (for more detail on the MD calculations refer to the Methods section). Furthermore, the influence of (Th,Pu)O_2_ actinide composition on oxygen diffusivity, oxygen defect energy and the superionic transition (also known as the Bredig transition and lambda transition) are also investigated. These properties have been previously investigated for (U,Pu)O_2_ and (U,Th)O_2_^13^,^14^,^15^,^16^,^17^.

## Results

### Thermal Expansion

**Figure 1:**
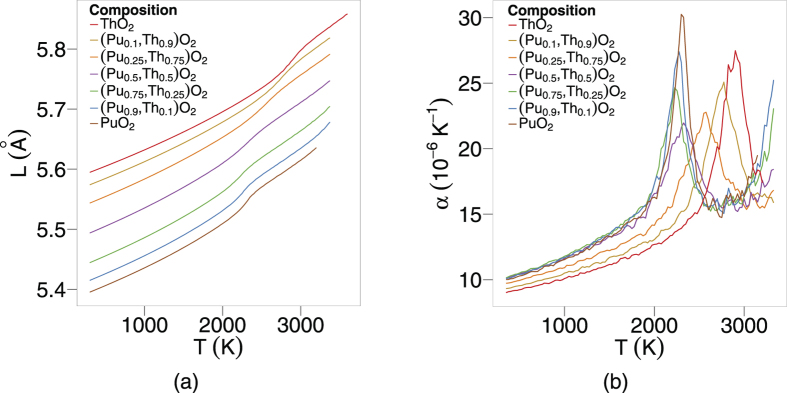
(**a**) Shows variation of lattice parameter as a function of temperature for eight compositions of (Pu_x_,Th_1−x_)O_2_. (**b**) Depicts the linear Thermal Expansion Coefficient as a function of temperature.

Lattice parameters were calculated by taking the cube root of the volume. The linear thermal expansion coefficient is then given by the relationship,


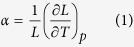


where L is the lattice parameter. The derivative of the lattice parameter with respect to temperature 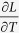 is calculated by fitting a straight line to the lattice parameter at a specific temperature and the points within 50 K on either side. The lattice parameter (L) as a function of temperature (T) is reported in Fig. 1a, averaged over the ten randomly generated structures for each mixed oxide composition.

The lattice parameter increases linearly with temperature until around 2200 K, where a small ‘bump’ occurs. This effect can be seen more clearly in a plot of the linear thermal expansion coefficient *α* as a function of temperature (Fig. 1b).

As will be described later (discussion section), the peaks seen in Fig. 1b correspond to the onset of the superionic transition. Although most of the peaks lie between the end member peaks, there is a skew towards the lower temperature peak exhibited by PuO_2_. Furthermore, small additions of Th^4+^ to PuO_2_ actually decreases the temperature of the peak. This behaviour has been observed previously for (U_x_,Pu_1−x_)O_2_ and (U_x_,Th_1−x_)O_2_^13^,^14^, however, in this previous work, the peaks for intermediate compositions were always between the bounds defined by the pure end members. In fact, for (U,PuO_2_) the skew was very small.

### Enthalpy and Specific Heat

**Figure 2:**
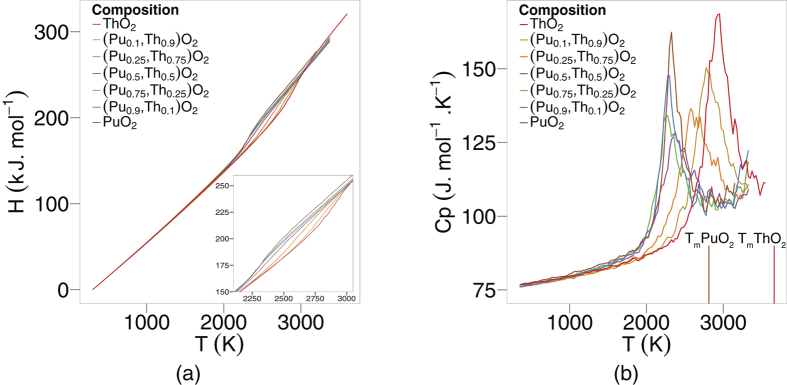
The variation of enthalpy increment as a function of temperature for for eight compositions of (Pu_x_,Th_1−x_)O_2_ is shown in (**a**). (**b**) Depicts the variation in specific heat capacity as a function of temperature, where T_m_ refers to the melting temperature depicted by the potential model^22^.

From the averaged lattice enthalpy, the enthalpy increment, H(T) - H(298 K), is determined per mol as a function of temperature (see Fig. 2a). The specific heat capacity, at a constant pressure can then be determined from the first derivative of the enthalpy:


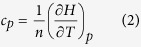


where *n* is the number of moles and 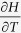 is calculated by fitting a straight line to the enthalpy increment at a specific temperature and the points within 50 K on either side, similarly to calculating 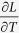. The specific heat as a function of temperature is reported in Fig. 2b.

As stated previously, the specific heat capacities in Fig. 2b for each composition grow steadily until about 2000 K, where they increase much more rapidly before decreasing, which gives rise to peaks. Similarly to Fig. 1b, the peaks for specific heat skew towards lower temperatures as the plutonium content is increased. Again, it is significant that the peaks for (Th_0.1_Pu_0.9_)O_2_ and (Th_0.25_,Pu_0.75_)O_2_ fall below the position of the pure PuO_2_ peak.

**Figure 3:**
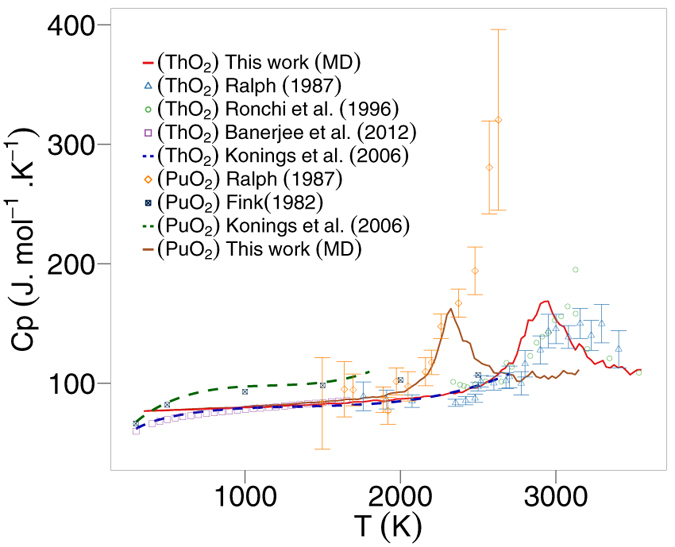
Variation of specific heat capacity against temperature for PuO_2_ and ThO_2_, where the solid lines represent MD results and the points represent experimental data^20^,^21^,^23^,^24^.

Experimental results for the specific heat capacities are plotted with the specific heat capacities predicted by MD for PuO_2_ and ThO_2_ in Fig. 3. The dashed lines are the recommended equations by Konings *et al*.^18^,^19^, however, these equations do not reproduce the heat capacity around the superionic transition^19^. Therefore, the equations have been cut off at 1800 K and 2700 K for PuO_2_ and ThO_2_, respectively. It is also notable that the PuO_2_ data by Fink^20^ does not exhibit a superionic transition. In Fink’s paper^20^ he acknowledges that “the high temperature PuO_2_ data were too scattered to determine whether a phase transition exists”. The results from Ralph^21^ were calculated using a quasi-local linear regression method on the enthalpy data. The choice of fitting method applied to enthalpy data to calculate specific heat values effects the outcome (values by Ralph^21^, Konings^18^,^19^ and Fink^20^ are calculated using the method of applying a fit through enthaply data). Thus, to ensure a fair comparison, polynomials should be fitted to the MD results that are consistent with the experimental data, as done by Cooper *et al*.^22^. Specific heat capacities of samples by Banerjee *et al*.^23^ were measured using heat flux type differential scanning calorimeters, while Ronchi *et al*.^24^ used a thermal arrest technique to conclude that a superionic transition occurs for ThO_2_. It has been suggested that new measurements for the specific heat capacity of PuO_2_ will be helpful^19^. Valu *et al*.^25^ measured the enthalpy of (Th,Pu)O_2_ as well as the end members as a function of temperature. The specific heat was obtained by using a simultaneous linear regression on the enthalpy values. Although the temperatures investigated in the paper did not consider the superionic regime, they did show a constant increase with temperature and are consistent with the MD data in this paper. For example, comparing the specific heat values for (Th_0.92_,Pu_0.8_)O_2_ and (Th_0.46_,Pu_0.54_)O_2_ of Valu *et al*.^25^ with the MD values for (Th_0.9_,Pu_0.1_)O_2_ and (Th_0.5_,Pu_0.5_)O_2_ respectively, over the temperature range 800–2000 K, the percentage difference varies from 0.7% to 9.6%. (Percentage values below 800 K were disregarded as empirical potentials cannot capture low temperature specific heat). It is important to note that the compositional difference between our MD data and the measurements of Valu *et al*. may contribute to the error.

### Oxygen Diffusivity

**Figure 4:**
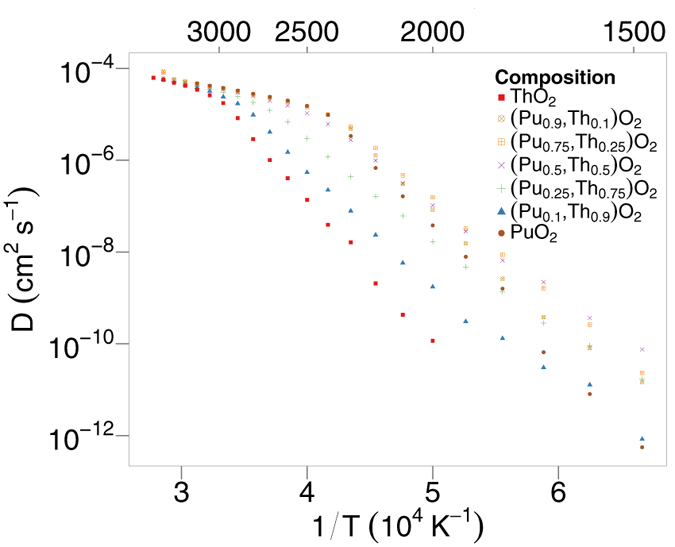
Oxygen diffusivity as a function of temperature for different compositions of (Pu_x_,Th_1−x_)O_2_, averaged over 10 randomly generated structures for each solid solution.

By plotting oxygen diffusivity *D* on a log scale as a function of 1/*T*, as presented in Fig. 4 (cation migration was not observed over the simulation timescale) and assuming an Arrhenius relationship the activation energy for oxygen migration (H_a_) can be obtained from the gradient of the graph:


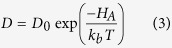


where *D*_0_ is the pre-exponential, *k*_*b*_ is the Boltzmann constant and *T* is the temperature.

It is interesting to note that Fig. 4 also shows non-Arrhenius behaviour, in that there is a change in gradient, which depends on composition. However, above and below this temperature there are two distinct linear regions that have a constant activation energy.

**Figure 5:**
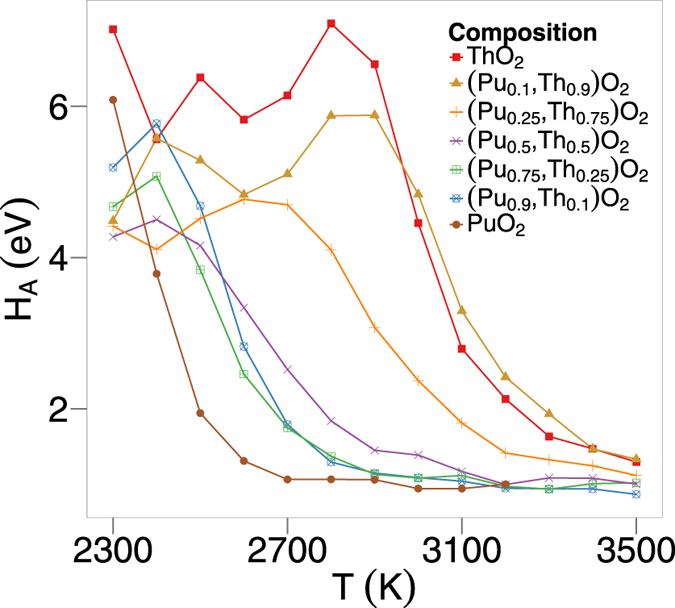
Activation energy, H_A_, as a function of temperature for oxygen migration in seven (Pu_x_,Th_1−x_)O_2_ compositions each averaged over 10 randomly generated structures.

Figure 5 depicts the activation energy as a function of temperature. This is calculated by taking the gradient of Fig. 4, in a similar manner to that of calculating the linear thermal expansion coefficient and the specific heat. From this it is clear that the activation energy starts high and drops to a much lower value in each case. For ThO_2_, the activation energy begins to drop at around 2750 K. Excluding (Th_0.9_,Pu_0.1_)O_2_, addition of Pu to ThO_2_ decreases the temperature at which the transition occurs. There is a clear correlation between the change in activation energy and the temperature at which peaks and bumps occur in Figs 1a to 2b.

**Figure 6:**
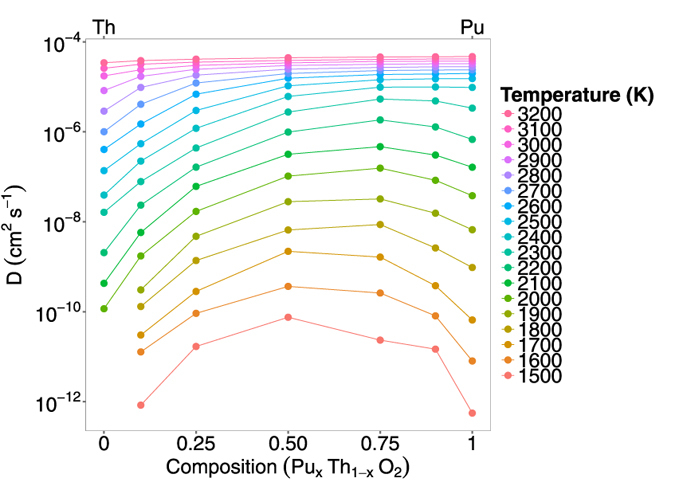
Oxygen diffusivity vs composition for (Pu_x_,Th_1−x_O_2_), for 10 randomly generated structures, averaged for each composition.

**Figure 7:**
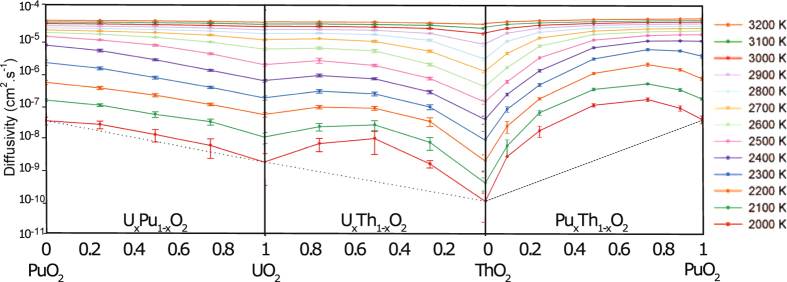
Oxygen diffusivity as a function of composition for (Pu_x_U_1−x_)O_2_, (U_x_Th_1−x_)O_2_ and (Pu_x_Th_1−x_)O_2_ for 10 randomly generated structures, averaged for each composition. The error bars correspond to the standard deviation of the 10 runs.

Figure 6 shows the effect of composition on oxygen diffusivity. At high temperatures (above 3000 K), the diffusion values across the entire compositional range are almost constant. In the temperature range 1700 K to 2400 K, diffusivity is greatly enhanced from the values of the pure systems. For the lowest simulation temperatures (below 1700 K) oxygen diffusivity is at a maximum for (Pu_0.5_,Th_0.5_)O_2_ and in the temperature range 1800 to 2700 K (Pu_0.75_,Th_0.25_)O_2_ exhibits the highest diffusivity. Moreover, the augmentation of oxygen diffusivity is greatest for lower temperatures, with the effect becoming reduced as temperature is increased. The effect seen in Fig. 6 is consistent with that of other solid solutions, (U_x_,Pu_1−x_)O_2_ and (U_x_,Th_1−x_)O_2_^13^,^14^,^16^ although enhanced diffusion is even more evident in (Th,PuO_2_) (see Fig. 7).

### Defect Energies

**Figure 8:**
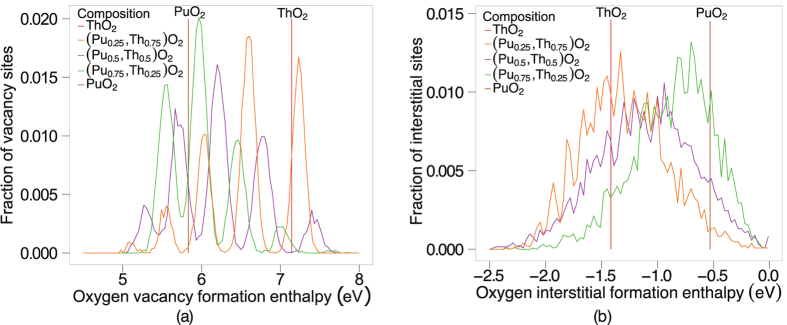
(**a**) The fraction of oxygen sites that lie within 0.005 eV of the corresponding oxygen vacancy formation enthalpy. The five peaks for each composition of (Th,Pu)O_2_ represent the five possible orientations of the first nearest cation neighbour surrounding the oxygen vacancy. From left to right, the peaks correspond to 4, 3, 2, 1, 0 Pu ions on nearest neighbour sites (or 0, 1, 2, 3, 4 Th ions). (**b**) Represents the fraction of oxygen sites that lie within 0.005 eV of the corresponding oxygen interstitial formation enthalpy.

Due to the large number of unique defect enthalpies, the oxygen vacancy defect formation energies shown in Fig. 8a have been binned in groups of width 0.01 eV, thereby showing the fraction of oxygen sites that lie within 0.005 eV of a given oxygen vacancy formation enthalpy. There are four first nearest neighbour cation sites surrounding the oxygen vacancy. Thus, the five distinct peaks of oxygen vacancy energies for each (Pu_x_,Th_1−x_)O_2_ composition correspond to the five possible configurations of the first nearest cation neighbours around the oxygen site (i.e 4, 3, 2, 1, 0 Pu cations or 0, 1, 2, 3, 4 Th cations), whereas the pure systems have a single value. Similarly to Fig. 8a, Fig. 8b shows the fraction of oxygen interstitials that lie within 0.005 eV of a given formation energy. There are a large number of defect enthalpies lower than the end members, regardless of composition. The enthalpy required to create oxygen vacancies underpins disorder on the sublattice and thus oxygen mobility. This is an important point and will be examined in the discussion section of this paper.

**Figure 9:**
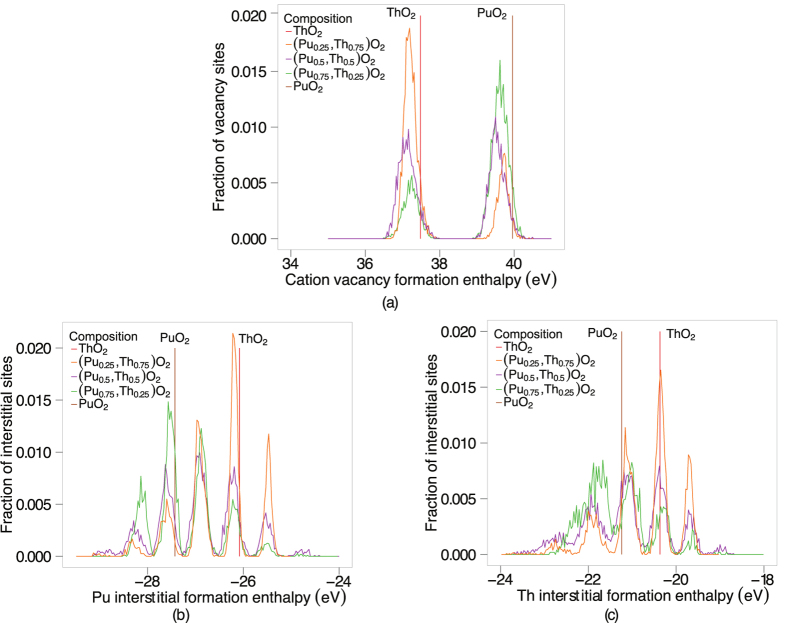
(**a**) The fraction of cation vacancy sites that lie within 0.005 eV of the corresponding cation vacancy formation enthalpy. There is one formation enthalpy for ThO_2_ and PuO_2_. Moreover, there is a perturbation of the pure peaks for the MOX systems as the distance to the nearest cation neighbour is too large to see distinct peaks. (**b**) Represents the fraction of Pu interstitial sites that lie within 0.005 eV of the corresponding Pu interstitial formation enthalpy while (**c**) represents the fraction of Th interstitial sites that lie within 0.005 eV of the corresponding Th interstitial formation enthalpy, respectively. The 7 peaks represent the 7 different ways of combining the Th, Pu ions surrounding an interstitial site.

Cation defect enthalpies are also examined and represented in Fig. 9a–c. Similarly to the oxygen defect formation enthalpies (seen in Fig. 8a,b), the defect formation enthalpies have been binned in groups of width 0.01 eV, thereby showing the fraction of cation sites that lie within 0.005 eV of a given cation defect formation enthalpy. Again, the pure systems have a single value. The MOX peaks closest to the end member value represents the cation of that particular species being removed to create a vacancy (see Fig. 9a). For instance, the peaks surrounding the ThO_2_ cation vacancy formation enthalpy represent vacancies on Th cation sites in MOX. Equivalently, the peaks surrounding the PuO_2_ cation vacancy formation enthalpy represent vacancies on Pu cation sites in MOX. This is also exemplified by the MOX peak heights in Fig. 9a. For example, (Pu_0.75_,Th_0.25_)O_2_ has the lowest peak height for Th cation vacancies and the largest peak for Pu cation vacancies. (Pu_0.5_,Th_0.5_)O_2_ has two peaks of similar sizes as there is an equal amount of Pu and Th cation vacancy sites.

There are six first nearest neighbour cation sites with respect to the interstitial. Therefore, in Fig. 9b,c, there are seven peaks for the cation interstitial formation enthalpies due to the seven distinct ways of ordering the Th and Pu cations (i.e 6, 5, 4, 3, 2, 1, 0 Pu cations or 0, 1, 2, 3, 4, 5, 6 Th cations). Again, for all MOX there are lower cation interstitial formation energies than for the end members.

**Figure 10:**
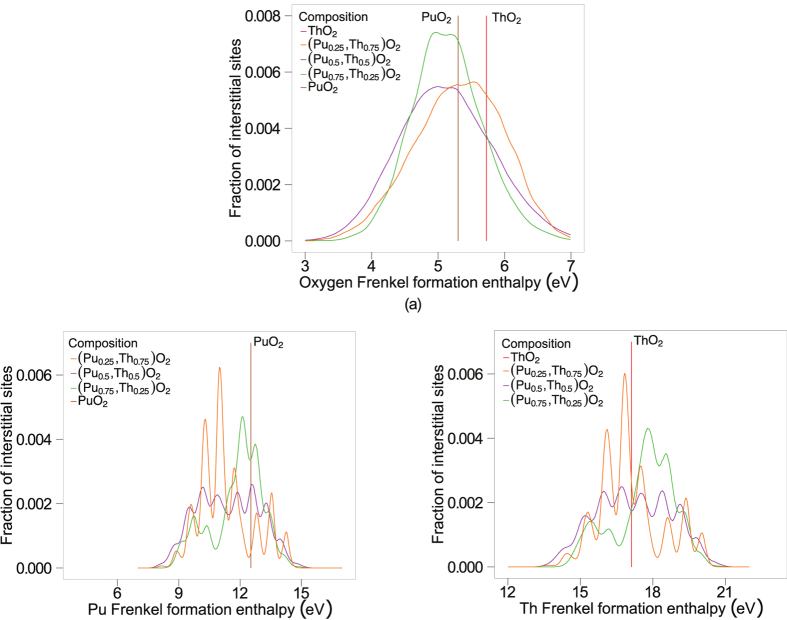
(**a**) By combining every possible combination of vacancy-interstitial pair the fraction of isolated Frenkel oxygen sites that lie within 0.005 eV of the corresponding oxygen Frenkel pair formation enthalpy is predicted. (**b**) Represents the fraction of Pu Frenkel pairs that lie within 0.005 eV of the corresponding Pu Frenkel formation enthalpy while (**c**) represents the fraction of Th Frenkel pairs that lie within 0.005 eV of the corresponding Th Frenkel formation enthalpy.

Oxygen Frenkel enthalpies were calculated by summing each value of the isolated oxygen vacancy formation energy with every isolated oxygen interstitial formation energy. These were binned in groups of width 0.01 eV and represented as a distribution, pictured in Fig. 10a.

Summation of the formation enthalpies for each isolated cation interstitial of a specific species with every isolated cation vacancy of the same species resulted in the cation Frenkel enthalpies for both Pu and Th cations. In other words, the Pu interstitial formation enthalpies have been summed with the peaks corresponding to the vacancies on Pu cation sites only, as a Pu interstitial with a Th vacancy is nonsensical. Likewise, the Th interstitial formation enthalpies have been summed with the peaks corresponding to the vacancies on Th cation sites only. Again, these have been binned in groups of width 0.01 eV and represented as a distribution (seen in Fig. 10b,c).

## Discussion

The thermophysical properties of (Th_x_,Pu_1−x_)O_2_ are, in part, associated with defect creation and oxygen diffusivity on the sublattice. As mentioned in the results section, there is a discrepancy from linear growth for the lattice parameter as a function of temperature (see Fig. 1a), where a small ‘bump’ is observed above 2000 K. The ‘bump’ can be seen more clearly as a peak in the plot of linear thermal expansion coefficient (Fig. 1b). Similarly, a ‘bump’ is seen in the enthalpy and a peak in the specific heat (Fig. 2a,b), which is commensurate with the ‘bump’ in the thermal expansion. This is associated with the creation of oxygen Frenkel defects and the superionic transition that the oxygen sublattice undergoes. Moreover, Fig. 5, is consistent with there being two contributions to the activation energy below the superionic transition. The formation energy for oxygen Frenkel pairs and the activation energy barrier for hopping. Above the transition the oxygen sublattice is saturated with defects, therefore the Frenkel pair formation energy no longer contributes and the activation energy decreases. However, since the lattice concentration of the defects is greater, the hopping energy will not necessarily be the same above and below the transition.

An enthalpy of formation is required to create defects and undergo the transition, which is evident in Fig. 2a,b. The oxygen defect formation energy is explained in terms of the change in lattice parameter and the oxygen vacancy defect volume. Compositions with a smaller Pu:Th ratio have a greater lattice parameter, which results in a downward shift in oxygen vacancy formation enthalpies (see Fig. 8a). As mentioned in the defect energies section, the five distinct peaks in Fig. 8a correspond to the five possible configurations of the first nearest cation neighbours around an oxygen site. Oxygen sites that are fully surrounded by Pu ions and in an environment with greater lattice parameter than PuO_2_, have lower oxygen formation enthalpies than the pure PuO_2_ system. Therefore, the (Pu_0.25_,Th_0.75_)O_2_ composition has the lowest oxygen vacancy defect peak as the oxygen site is surrounded by small Pu ions and the lattice parameter of (Pu_0.25_,Th_0.75_)O_2_ is greater than PuO_2_. Nonetheless, the peak height is small due to the low number of Pu cations. The creation of a defect, when introduced into the lattice causes a distortion in its surroundings resulting in a volume change. Consequently, lattice swelling due to the creation of oxygen defects results in peaks in the linear thermal expansion coefficient. It is also interesting to note, Figs 1b and 2b shows that for ThO_2_ the peaks in linear thermal expansion coefficient and specific heat are at around 2950 K, in close agreement to the experimental value of 3090 K^24^. Above the transition temperature the oxygen lattice is in effect saturated with defects and as no additional oxygen disorder can be created, the specific heat and linear thermal expansion coefficient decrease. Also, the apparent onset of a second peak is probably due to the creation of significant concentrations of cation defects. However, this is not significant below the melting point predicted by the potential.

The transition temperature is dependent on the composition for (Th_x_,Pu_1−x_)O_2_, which is evident in Figs 1b and 2b. The superionic transition temperature is highest in ThO_2_, but rather than the lowest occurring in PuO_2_, and (Th,Pu)O_2_ being a linear interpolation of the two, there is a skew of the superionic transition peaks to lower temperatures, with (Pu_0.9_,Th_0.1_)O_2_ and (Pu_0.75_,Th_0.25_)O_2_ occurring slightly lower than PuO_2_. In fact, only the addition of 75% and 90% Th to PuO_2_ causes the superionic transition to increase significantly. This indicates that the cation composition in (Th,PuO_2_) affects the thermal equilibrium concentration of O defects through the formation energies, something that will be discussed later.

Underpinning the superionic transition is the creation and mobility of oxygen defects. Figure 4 shows two distinct regions with constant gradients (and therefore activation energies), suggesting two diffusion regimes, where there is a change in the diffusion mechanism at high temperature, after the superionic transition. Using the gradients from Figs 4 and 5 demonstrates that there is a decrease in activation energy as the system undergoes the superionic transition. After the superionic transition the structure becomes saturated with oxygen defects, enabling the oxygen sublattice to exhibit a highly disordered structure, thus explaining the change in diffusion regime.

Below the superionic transition, Fig. 6 indicates that addition of Th to Pu actually enhances diffusion despite ThO_2_ exhibiting lower diffusion than PuO_2_. As the temperature is decreased the enhancement is more extreme, with the enhanced diffusivity having higher values than the end members. Comparing the diffusivity of (Pu_x_,Th_1−x_)O_2_ to (Pu_x_,U_1−x_)O_2_^14^ and (U_x_,Th_1−x_)O_2_^13^, it can be seen that the enhancement is greatest for (Pu_x_,Th_1−x_)O_2_. It is also interesting to note, that although ThO_2_ has a much lower diffusivity than UO_2_, additions of Th to PuO_2_ enhance the diffusivity more than adding U to PuO_2_. The enhanced diffusivity is a consequence of the oxygen defect formation enthalpy. For the oxygen vacancy formation enthalpy (Fig. 8a), depending on the the ratio of Pu:Th the probability of a certain coordination environment changes: the lowest enthalpy peaks commensurate with higher coordination by Pu ions and vice versa for the highest enthalpy peaks with Th ions. This is unsurprising as the oxygen formation energy is lower for PuO_2_ than ThO_2_. Below the superionic transition, the oxygen defect formation enthalpy is part of the activation energy, as it is needed to create oxygen defects. At low temperature the enthalpy peaks in Fig. 8 are more important. The MOX has lower enthalpy peaks than PuO_2_ and therefore higher defect concentrations at lower temperatures. As a consequence, below the superionic transition, enhanced diffusion is seen for MOX relative to PuO_2_ (Fig. 6). As the temperature increases higher enthalpy peaks/defects are accessible until the lattice becomes saturated. This occurs up to the superionic transition where the system is saturated with oxygen defects and the activation energy decreases.

The superionic transition only consists of oxygen defects and this is clarified in Figs 9 and 10. The cation defect formation enthalpies are too high to activate at the superionic transition temperatures.

Applying the logic from the oxygen defect formation enthalpy to the cation Frenkel formation enthalpies in Fig. 10b,c we notice that the MOX formation energies are lower than the pure members, suggesting that there can be an enhancement for MOX cation diffusion at lower temperatures. This can be significant for fission product mobility and it may also effect the melting temperatures of MOX^26^.

### In Summary

MD simulations were performed to investigate the superionic transition for different compositions of (Th,Pu)O_2_, including PuO_2_ and ThO_2_. The thermal dependence of lattice parameter, linear thermal expansion coefficient, enthalpy and specific heat at constant pressure for (Th,Pu)O_2_ systems all show features that are a consequence of the superionic transition. This work suggests the superionic transition is associated with Frenkel oxygen defects, agreeing with work carried out previously^24^,^27^,^28^,^29^. (Although, there are suggestions that electronic disorder makes an important contribution to the peak in heat capacity it is not clear over which temperature ranges this effect would dominate^30^,^31^). The transition can also be identified from a change in oxygen activation energy and thus a change in the oxygen diffusion mechanism. Below the superionic transition, there is an increase in oxygen diffusivity for (Th_x_,Pu_1−x_)O_2_ compared to the pure end members ThO_2_ and PuO_2_. This increase in oxygen diffusivity is a consequence of lower oxygen defect formation energies for (Th_x_,Pu_1−x_)O_2_ than that of the pure systems. Comparison of oxygen diffusivity for (Th_x_,Pu_1−x_)O_2_ compared to (Pu_x_,U_1−x_)O_2_ and (U_x_,Th_1−x_)O_2_ shows that (Th_x_,Pu_1−x_)O_2_ exhibits the greatest enhancement. This highlights the importance of the lattice parameter mismatch between the end members in that (Th,Pu)O_2_ has the greatest mismatch, a point mentioned previously^14^. The creation of oxygen defects induces a volume change on the system, which demonstrates why the superionic transition is responsible for lattice expansion at higher temperatures, while the energy needed to create these oxygen defects causes the rise in enthalpy and a peak in the specific heat, both as a function of temperature. The much higher cation vacancy and interstitial formation energies explain why the superionic transition is a consequence of oxygen disorder only.

## Methods

Interatomic forces were described using the Cooper, Rushton and Grimes (CRG) potential model^22^. This combines effective pair-potentials with the many-body EAM description of Daw and Baskes^32^. It has been shown previously to describe the bulk modulus, lattice parameters, specific heat, enthalpy and elastic constants of a variety of actinide oxides from 300 to 3500 K^22^. Additionally, the coulombic interactions between ions were included. These used partial charges and were calculated using a particle mesh method^33^. Based on a previous study, an updated version of this potential model has been used for Pu interactions^14^. The potential is robust at high temperatures and is in reasonable agreement with the melting points (T_m_) of UO_2_, PuO_2_ and ThO_2_^13^,^14^,^22^. The accurate description of the melting point makes it particularly suitable for investigating the superionic transition, as has been demonstrated for (U_x_,Pu_1−x_)O_2_ and (U_x_,Th_1−x_)O_2_^13^,^14^. This is of particular importance as the superionic transition occurs at 0.85T_m_ in fluorite structures^27^,^34^,^35^. In fact, many potential models predict the superionic transition as shown by Potashnikov *et al*.^15^,^16^.

MD simulations were implemented using the Large-scale Atomic/Molecular Massively Parallel Simulator (LAMMPS)^36^. In order to reduce the effect of bias introduced by any particular configuration, ten distinct, random distributions, were generated for each composition. The results that follow were obtained by taking averages over these ten configurations. Simulations were performed for (Th_x_,Pu_1−x_)O_2_, with x = 0, 0.1, 0.25, 0.5, 0.75, 0.9 and 1. Systems were generated by randomly assigning Th^4+^ and Pu^4+^ to the cation sites in a perfect 10 × 10 × 10 fluorite supercell to the desired composition. This random assignment of cations was carried out for each structure independently. Following the creation of the structure, static energy minimisation was used to reduce the effects that local energetic configurations, introduced by random assignment, may have on the subsequent MD runs. MD calculations were performed using a sequential temperature ramp with steps of 25 K from 300 K to 3200 K. At each temperature the system was equilibrated for 40 ps with the volume and enthalpy averaged over the final 2 ps. This allowed the system dimensions and enthalpy to be determined as a function of temperature. For all the calculations, a timestep of 1 fs and a short-range potential cut-off of 11 Å were employed. A Nosé-Hoover thermostat and barostat^37^,^38^ were applied for simulations in the constant pressure-temperature (NPT) ensemble, and these used relaxation times of 0.1 ps and 0.5 ps, respectively.

To determine the oxygen diffusivity the structures were first equilibrated at the temperature of interest for 25 ps in the NVT ensemble using the volumes previously calculated. Subsequently, the oxygen MSD was determined for 1 ns in the NVE ensemble. Diffusivity (D) is related to the mean square displacement 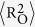 via the Einstein relationship^39^:


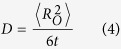


where t represents the simulation time and 6 is the number of directions diffusion may occur.

For the isolated oxygen/cation vacancy and interstitial defect formation enthalpy calculations, the same supercells described previously were implemented. To ensure the local energy minima within the non-defective structures are found, energy minimisation consisted of three steps. Firstly, the structure was minimised using a damped dynamics algorithm^40^ under constant volume conditions, followed by a conjugate gradient method, where the simulation box dimensions were allowed to vary at a constant pressure step, before a final optimisation employing a steepest decent procedure^41^,^42^ with fixed lattice parameters. Point defects were then introduced into the structures by either removing oxygen ions from their sites (vacancy) or placing oxygen ions at interstitial positions. Using fixed lattice parameters, the defective supercells are energy minimised to represent the dilute limit. The defect enthalpies, dH, are given by:


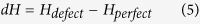


where the total enthalpies for the defective and perfect supercells are given by H_defect_ and H_perfect_, respectively. The oxygen Frenkel enthalpy for ThO_2_ and PuO_2_ for the 10 × 10 × 10 supercell are converged within 0.1 eV of the fully isolated enthalpies given by Cooper *et al*.^22^. Therefore, defect enthalpy convergence with respect to the system size is assumed and the dilute limit criterion satisfied.

As mentioned previously, oxygen vacancies are created by removing oxygen ions from lattice sites. This is done for each of the 10 configurations, leading to 80,000 defect simulations for each composition. Likewise, 40,000 oxygen interstitial defect simulations were created by placing oxygen ions at interstitial sites, for each composition. A similar approach was undertaken to calculate cation vacancies and interstitials.

## Additional Information

**How to cite this article**: Galvin, C. O. T. *et al*. Thermophysical properties and oxygen transport in (Th_x_,Pu_1−x_)O_2_. *Sci. Rep.* **6**, 36024; doi: 10.1038/srep36024 (2016).

**Publisher’s note:** Springer Nature remains neutral with regard to jurisdictional claims in published maps and institutional affiliations.
